# Effect of hot water immersion on acute physiological responses following resistance exercise

**DOI:** 10.3389/fphys.2023.1213733

**Published:** 2023-07-05

**Authors:** Joshua S. Jackman, Phillip G. Bell, Ken Van Someren, Marcela B. Gondek, Frank A. Hills, Laura J. Wilson, Emma Cockburn

**Affiliations:** ^1^ London Sport Institute, Middlesex University, London, United Kingdom; ^2^ Art Health Solutions, Newcastle, United Kingdom; ^3^ Department of Sport, Exercise and Rehabilitation, Faculty of Health and Life Sciences, Northumbria University, Newcastle, United Kingdom; ^4^ Sports Lab Northwest, Atlantic Technological University, Donegal, Ireland; ^5^ Biomarker Research Group, Department of Natural Sciences, Middlesex University, London, United Kingdom; ^6^ School of Biomedical Sciences, Newcastle University, Newcastle, United Kingdom

**Keywords:** heat therapy, strength training, temperature, recovery, muscle damage, inflammation

## Abstract

**Purpose:** Hot water immersion (HWI) is a strategy theorised to enhance exercise recovery. However, the acute physiological responses to HWI following resistance exercise are yet to be determined.

**Methods:** The effect of HWI on intramuscular temperature (IMT), muscle function, muscle soreness and blood markers of muscle cell disruption and inflammatory processes after resistance exercise was assessed. Sixteen resistance trained males performed resistance exercise, followed by either 10 min HWI at 40°C or 10 min passive recovery (PAS).

**Results:** Post-intervention, the increase in IMT at all depths was greater for HWI compared to PAS, however this difference had disappeared by 1 h post at depths of 1 and 2 cm, and by 2 h post at a depth of 3 cm. There were no differences between groups for muscle function, muscle soreness or any blood markers.

**Conclusion:** These results suggest that HWI is a viable means of heat therapy to support a greater IMT following resistance exercise. Recovery of muscle function and muscle soreness is independent of acute changes in IMT associated with HWI.

## Introduction

Post-exercise hydrotherapy is common practice among athletic individuals, with the goal of enhancing acute recovery following training and competition (Vaile et al., 2010). While cold water immersion has received growing attention in the literature, there is a paucity of research investigating the effectiveness of hot water immersion (HWI) on exercise recovery in humans, with equivocal findings to date (McGorm et al., 2018). The efficacy of such strategies is likely related to the specific recovery needs of the individual (Minett and Costello, 2015), therefore a greater understanding of the impact of HWI on acute physiological responses will aid in the application of this strategy in exercise recovery.

HWI is thought to exert a physiological impact primarily through an increase in cutaneous and subcutaneous tissue temperature which induces peripheral vasodilation and a subsequent increase in blood flow (Wilcock et al., 2006). The ensuing increase in permeability of cellular, lymphatic and capillary vessels may drive an increased rate of metabolism, nutrient delivery and clearance of waste products (Coté et al., 1988; Baker et al., 2001), which could aid exercise recovery. The research behind these proposed mechanisms has typically occurred in the field of physiotherapy or with techniques of heat application including ultrasound and heat packs (Wyper and McNiven, 1976; Knight and Londeree, 1980; Bonde-Petersen et al., 1992). Whether similar effects would be seen with HWI and following an exercise session which stimulates acute physiological responses typical of a ‘real world’ training session requires further investigation.

Despite the limited insights into the mechanisms that would rationalise enhanced recovery in an exercise setting, research from human studies using post-exercise heating has produced some promising evidence (Vaile et al., 2010; McGorm et al., 2018). Cheng et al. (2017) reported that acute recovery following exhaustive intermittent arm cycling is influenced by intramuscular temperature, with mean power output better preserved after the upper limbs were heated to ∼38°C compared to being cooled to ∼15°C for 2 h post-exercise. Based upon replication of these findings in an animal model, the authors attributed the enhanced recovery to better rates of glycogen resynthesis with heating versus cooling, due to the increased tissue temperature promoting rates of enzymatic processes (Cheng et al., 2017). Others have also reported HWI to improve the recovery of strength (Clarke, 1963; Vaile et al., 2008) and power (Viitasalo et al., 1995) following fatiguing isometric exercise, a leg press protocol designed to elicit delayed onset-muscle soreness and throughout a strength/power training week for track and field athletes, respectively. Enhancing recovery in this way would be of benefit to those performing resistance exercise as part of a progressive programme, however the effect of HWI on these measures following an ecologically valid exercise session are yet to be determined.

The effect of post-exercise heating on other acute physiological responses is inconclusive. Following high-intensity intermittent exercise (Pournot et al., 2011) and an intense strength/power training week (Viitasalo et al., 1995), HWI did not influence the appearance of intramuscular enzymes in the blood. Whereas after a single bout of eccentric exercise, Vaile et al. (2008) reported a reduction in plasma creatine kinase with HWI. Heat therapy may reduce pain via an analgesic effect on nerves (Baker et al., 2001) and has been shown to reduce soreness following lumbar extension exercise (Mayer et al., 2006). Conversely, other reports have demonstrated post-exercise heating to exert no impact upon muscle soreness (Viitasalo et al., 1995; Kuligowski et al., 1998; Vaile et al., 2008; Pournot et al., 2011). Differences in exercise modality, HWI protocol and timing of recovery measures make conclusions problematic, lending to the assertion that further research utilising ecologically valid protocols are required in this area (Vaile et al., 2010; McGorm et al., 2018).

A disparity currently exists between the hypothesised benefits of post-exercise heating and the evidence for HWI to improve acute exercise recovery (Vaile et al., 2010; McGorm et al., 2018). Given the growing use of thermotherapy in recreationally active and athletic populations, further insights are required to provide evidence-based rationale supporting or refuting the application of HWI as a recovery aid. Therefore, the aim of the present study was to investigate the effect of HWI on a range of acute physiological responses following resistance exercise.

## Methods

### Participants

Sixteen strength trained males volunteered to take part in the study (Table 1). Participants were considered to be resistance-trained if they had performed ≥3 resistance sessions per week for ≥2 years with a minimum of one session per week including exercises that targeted the lower limbs (Buckner et al., 2017). Prior to any experimental procedures, written informed consent was obtained from all individual participants and the study conformed to the latest revision of the Declaration of Helsinki (World Medical Association, 2013). All participants completed a health screening questionnaire and were excluded from the study if the investigator deemed they were contraindicated to the study procedures. The study was granted ethical approval (application number: 1,686) by the London Sports Institute Ethics Sub-Committee at Middlesex University.

**TABLE 1:** Participant characteristics.

	Intervention group	
	PAS (*n* = 8)	HWI (*n* = 8)
Age (yrs)	24 ± 4	25 ± 4
Height (m)	1.77 ± 0.05	1.80 ± 0.07
Body mass (kg)	88 ± 17	89 ± 14
Surface area:body mass	32 ± 3	32 ± 4
1 RM (kg)	158 ± 30	158 ± 31

PAS, passive recovery group; HWI, hot water immersion group; RM, repetition maximum.

### Experimental design

Using a between-subject design, participants were pair matched for baseline strength (1 repetition maximum [RM] back squat), and body composition (body surface area to body mass ratio) in line with previous research (Roberts et al., 2015) and assigned to HWI (*n* = 8) or passive recovery (PAS) (*n* = 8) groups. Previous research has shown the relationship between body surface area relative to body mass to be an important influencer on thermal and physiological responses to hydrotherapy (Stephens et al., 2014). Participants attended the laboratory on four occasions; during the first visit, anthropometric data was collected (height and body mass), before participants were familiarised with experimental procedures. Additionally, participants performed a strength assessment to determine a 6 RM for the exercise techniques used in the exercise session (back squat, front squat, good morning, Bulgarian split-squat). The second visit involved a body composition assessment prior to a further familiarisation with experimental procedures. Participants then performed a strength assessment to determine a 1 RM for the back squat.

**FIGURE 1:**
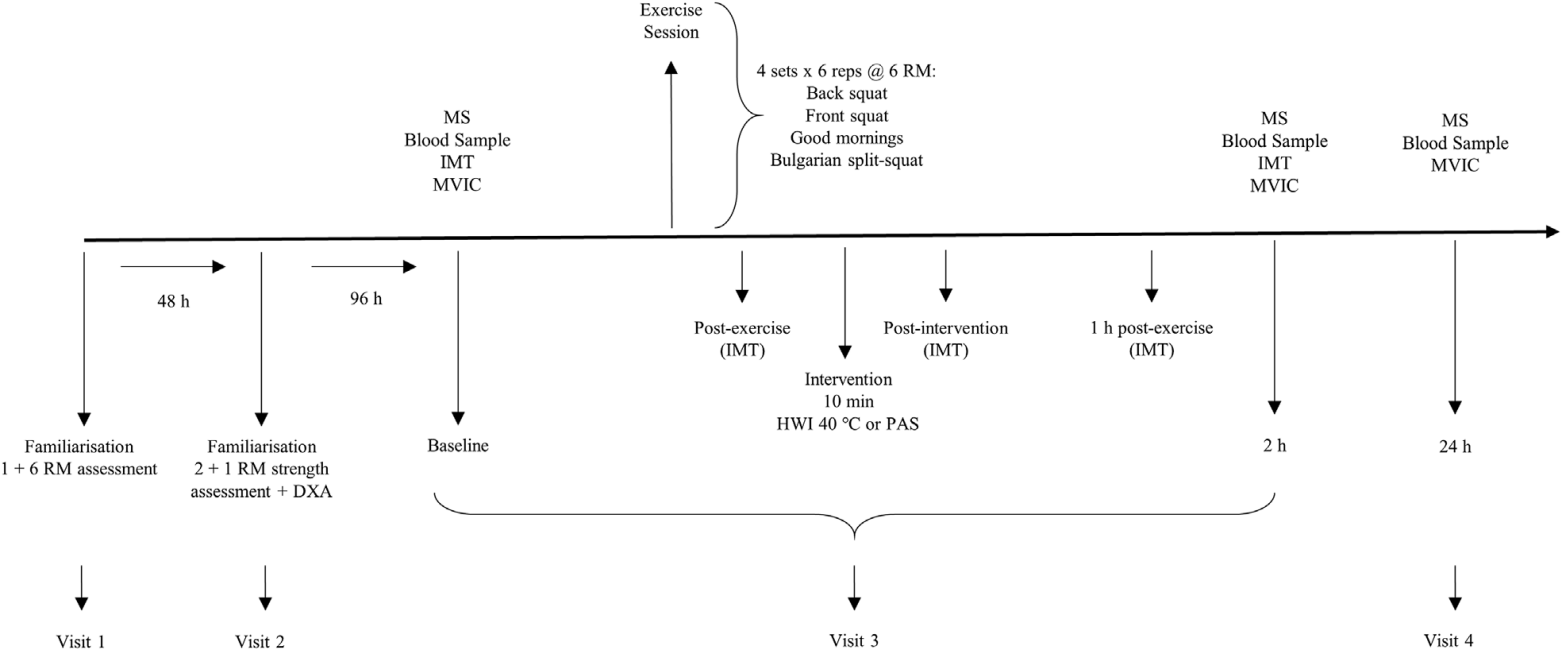
Schematic overview of study timeline. The order of assessments is depicted for the Baseline session. MS, muscle soreness; IMT, intramuscular temperature; MVIC, maximal voluntary isometric contraction; RM, repetition maximum; DXA, dual-energy x-ray absorptiometry; HWI, hot water immersion; PAS, passive recovery.

Visit 3 formed the start of the experimental period and required participants to perform baseline assessments in the following order: muscle soreness, blood sample, intramuscular temperature and maximal voluntary isometric contraction (MVIC). Following baseline assessments, participants performed a resistance exercise session and then completed either the HWI or PAS interventions. Intramuscular temperature assessments were repeated: post-exercise, post-intervention, 1 h and 2 h post-exercise. Muscle soreness, blood sample and MVIC were repeated at 2 h post-exercise. During visit 4, participants returned to the laboratory to perform 24 h post-exercise assessments for muscle soreness, blood sample and MVIC (Figure 1).

Prior to visit 3, participants were provided with a standardised meal (27 g porridge oats, 180 mL semi-skimmed milk, 200 g high protein yoghurt) to consume 2 h before arrival at the laboratory. Participants also consumed a ready to drink protein milk (30 g protein) following completion of the exercise session and after the 2 h post-exercise assessments, between which they were required to be fasted (Roberts et al., 2015). Additionally, participants were asked to refrain from food consumption in the 2 hours prior to any testing procedures. Aside from the control measures, participants were instructed to maintain their habitual dietary intake throughout the study. Participants were required to avoid the following throughout the study: exercise external to the protocol, any therapeutic interventions or nutritional supplements, alcohol and non-steroidal anti-inflammatory drugs.

### 6 RM strength assessment

Strength assessments were performed using a 6 RM testing protocol in accordance with recognised guidelines (Haff and Triplett, 2015). Following a standardised warm-up (Table 2), participants performed three sets of six repetitions of back squat with a progressively increasing load that corresponded to 50, 75% and 90% of their perceived 6 RM. Participants then performed sets of six repetitions with an increasing load for the determination of 6 RM with 2 min rest afforded between attempts. All 6 RM determinations were made within four attempts which were deemed successful by an investigator if a participant had reached a position in which the thigh was at least parallel to the floor. Participants repeated the above procedure for the determination of 6 RM on the front squat, good mornings and Bulgarian split-squat exercises with successful attempts determined by an investigator against standardised techniques. During the Bulgarian split-squat, participants placed the top of the toes of the trail leg on a 12 inch platform (McCurdy et al., 2010). The lead leg was placed approximately 39–45 inches from the front edge of the platform supporting the trail leg. Participants were then required to squat to a depth where the thigh of the lead leg was parallel to the ground before returning to the start position. These exercises were chosen to target a range of lower limb musculature and are commonly included in strength and conditioning programmes (Haff and Triplett, 2015). All resistance exercises were performed using free weights and a standard 20 kg bar (ELEIKO SPORT, Illinois, United States).

**TABLE 2:** Standardised warm up.

Exercise	Distance/Repetitions
Forward/backward shuttle run	10 m x 3 reps
Side to side shuttle	10 m x 3 reps
Forward/backwards lunge	10 m x 1 rep
Inch worms into spiderman	5 reps
Single leg RDL with high knee pull	5 reps each side
Bodyweight squat	5 reps
Glute bridge into heel walkouts	5 reps
Single leg glute bridge	5 reps each side
Jump squats	5 reps

RDL, Romanian deadlift.

### 1 RM strength assessment

For the purposes of matching groups for baseline strength, maximal lower-body strength was assessed by 1 RM testing in the back squat consistent with recognized guidelines established by the National Strength and Conditioning Association (Baechle and Earle, 2008).

### Resistance exercise session

Following the standardised warm up (Table 2), participants performed three sets of six repetitions of back squat with a load corresponding to 50, 75% and 90% 6 RM. Participants then performed four sets of six repetitions with a load corresponding to 6 RM for the following exercises: back squat, front squat, good mornings and Bulgarian split-squat. The intensity (100% 6 RM or ∼85% 1 RM) and volume (12 sets targeting the quadriceps muscle group) of the session were selected based upon recommendations that loads of 80%–95% 1 RM elicit maximal gains in strength (Peterson et al., 2005), and hypertrophy (Fry, 2004). The performance of at least 8–10 weekly sets per muscle group has also been suggested to be required to maximise increases in muscle strength (Peterson et al., 2005) and size (Schoenfeld et al., 2016) in trained individuals. Participants were instructed to perform the eccentric phase of the exercises in a controlled fashion lasting approximately 2 s, whilst the concentric phase was to be performed with maximal acceleration. This method of lifting was chosen given the suggestion that it is the intended rather than the actual velocity that determines the velocity-specific training response (Behm and Sale, 1993). Two minutes rest was afforded between sets and exercises, which has been recommended as a minimum for maximising gains in muscle size (Schoenfeld et al., 2015).

### Interventions

The HWI or PAS interventions were performed within 10 min post-completion of the resistance exercise session. Participants in the HWI group sat in an inflatable bath (iSprint, iCoolsport, Miami, Australia) and were required to submerge their legs in the water up to their waist in a seated position (hip angle of ∼90°), with their legs outstretched and relaxed. Water temperature was maintained at 40°C using a circulatory heating unit (iCool dual temperature LITE, iCoolsport, Miami, Australia) and participants were required to remain immersed for 10 min. Participants in the PAS group sat on a physiotherapy bed and were required to remain still in a seated position (hip angle of ∼90°), with their legs outstretched and relaxed for 10 min. To avoid any external confounding influences on temperature, all participants were required to remain in the laboratory until after all the assessments were complete on visit 3.

### Body composition

In order to match groups for body surface area to body mass ratio, body composition was assessed by dual-energy x-ray absorptiometry (DXA; GE Lunar Prodigy, GE Healthcare, Bucks, United Kingdom) in accordance with previous research (Bell et al., 2016).

### Intramuscular temperature

Intramuscular temperature was measured using a re-useable sterile needle thermistor (MKA08050-A, Ellab A/S, Rodovre, Denmark) with data read via a thermocouple system (E-Val Flex, Ellab A/S, Rodovre, Denmark). Previous studies have reported precision of 0.1°C for this system (Mohr et al., 2004). The needle thermistors were sterilised using an autoclave prior to each use according to manufacturer guidelines. The site of insertion was identified as the mid-point of the vastus lateralis muscle of the right limb, between the superior border of the patella and the inguinal fold and was marked using a pen for consistency between assessments with the site of insertion sterilised using a topical antiseptic (Betadine, Purdue Products LP, CT, United States). To ensure consistency in the depth of insertion, skin thickness and adipose tissue was measured using a caliper and a piece of medical tape was placed from the end of the needle at a distance which corresponded to 3 cm plus half of the skinfold thickness. The needle was then inserted into the vastus lateralis muscle until the tape contacted the skin surface, ensuring an intramuscular temperature of 3 cm. Once the reading had stabilised (approximately 2 s), the temperature was recorded. The needle was then manually removed to a depth of 2 cm and the temperature recorded once the reading had stabilised. This process was repeated for a depth of 1 cm prior to the needle being removed. Previous research has used this method to determine the effect of water immersion on intramuscular temperature following resistance exercise (Mawhinney et al., 2017).

### Maximal voluntary isometric contraction

Participants were seated on the dynamometer chair (Biodex 3, Biodex Medical Systems, NY, United States) with a hip joint angle of 90° and a knee joint angle of 70° (Eddens et al., 2017), set by the investigator using a goniometer. A knee joint angle of 70° has been shown to be sensitive to detect reduced muscle function following eccentric exercise, with no difference between this angle and the torque produced at 90° (McHugh and Tetro, 2003). Participants completed a standardised warm-up consisting of efforts at 50, 75% and 90% of perceived maximal force. Participants then performed three maximal voluntary isometric contractions (MVIC) of the right limb, each lasting 3 s, with standardised verbal instruction and encouragement provided throughout. Sixty seconds rest was afforded between attempts with peak force (N) recorded and the best attempt used for subsequent analysis.

### Active muscle soreness

Active muscle soreness was determined using a 200 mm visual analogue scale (VAS) with “no pain” indicated at one end and “pain/soreness as bad as it could be” at the other (Bell et al., 2014). Participants were instructed to stand with hands on hips and feet shoulder width apart prior to performing a squat to a depth whereby the thigh was parallel to the floor. Upon completion, participants indicated the pain felt in the lower limbs by drawing a line on the VAS.

### Blood sample collection and analysis

Venous blood samples were collected using the venepuncture technique from a vein in the ante-cubital fossa region by a trained phlebotomist. Blood was collected into two 5 mL serum separator tubes and left to clot for 30–60 min prior to being centrifuged at 3,000 *g*, 23°C for 8 min. The serum was then removed and immediately stored in aliquots at −80°C for later analysis. Blood samples were analysed for markers of: muscle cell disruption (creatine kinase [CK]) and inflammatory processes (interleukin-6 [IL-6], high-sensitivity c-reactive protein [hsCRP], matrix metalloproteinase-9 [MMP-9]). The time of collection for all blood markers was based upon likely known time-course responses, and peak changes post-exercise (Table 3).

**TABLE 3:** List of blood markers and associated time points of collection.

Marker	Baseline	2 h	24 h
CK	✓		✓
hs-CRP	✓		✓
IL-6	✓	✓	✓
IL-10	✓	✓	✓
MMP-9	✓	✓	✓

CK, creatine kinase; hs-CRP, high-sensitivity c-reactive protein; IL-6, interleukin-6; IL-10, interleukin-10; MMP-9, matrix metalloproteinase-9.

Serum CK and hs-CRP were determined by electrochemiluminescence using an automated analyser (Roche c702 chemistry module, Roche Diagnostics Ltd., United Kingdom). Serum IL-6 (Invitrogen Corporation, California, United States, MAN0006706) and MMP-9 (Thermo Scientific, Maryland, United States, BMS 2016-2 and BMS 2016-2TEN) were determined by an enzyme-linked immunosorbent assay (ELISA) using commercially available kits.

### Statistical analysis

Raw data is reported as mean ± SD. All data (except soreness) were checked for normality using Shapiro-Wilk, which is recommended for sample sizes less than 30. Data identified as non-normal was log-transformed and all further data analysis was conducted on the transformed data. A mixed model ANOVA was used to analyse each dependent variable with a between-subjects factor of group (HWI and PAS) and a within-subject factor of time (baseline and post resistance exercise timepoints). Mauchley’s test of sphericity was used to assess the homogeneity of variance and, where necessary, Greenhouse-Geisser corrections were applied. Significant main and interaction effects were analysed using Bonferonni *post hoc* pairwise comparisons. Partial eta squared (ηp^2^) was used to indicate the effect sizes for main and interaction effects with ≥ 0.01, ≥ 0.059, ≥ 0.138 indicating small, moderate and large effects, respectively (Cohen, 1988). SPSS (IBM Corp., IBM SPSS Statistics for Windows, Version 28, Armonk, NY) was used for statistical analysis with a significance level of *p* < 0.05.

The mean ± 95% confidence interval (CI) for the differences between groups for changes between baseline and post measurement points were calculated (HWI minus PAS group). ‘Unclear’ was used to describe 95% CI’s that crossed 0. Cohen’s *d* effect sizes were used to determine the magnitude of the difference between the groups at each time point with < 0.20 indicating a trivial effect, 0.20—0.49 a small effect, 0.50—0.79 a medium effect, 0.80—1.19 a large effect, and ≥ 1.2 a very large effect (Cohen, 1988).

## Results

The average (mean ± SD) values for each dependent variable for both groups at all time points are shown in Table 4. The same volume load was performed in the exercise session by both groups, with no significant differences seen between groups for any of the exercises. Post hoc analyses using the group means for post-intervention IMT at 1 cm depth, determined an effect size of 0.92 with an alpha level of 0.05, and yielded a statistical power of 1.00.

**TABLE 4:** Mean and SD for all dependent variables at each time point in both HWI and control.

Variable	Time point	HWI	PAS	Variable	Time point	HWI	PAS
MVIC (N)	Baseline	258 ± 99	226 ± 39	IMT at 1 cm (°C)*^$^	Baseline	33.52 ± 0.76	33.17 ± 1.18
	2 h*	214 ± 76	196 ± 49		P-ex	34.60 ± 1.51	33.95 ± 1.02
	24 h*	235 ± 79	212 ± 45		P-int	35.00 ± 0.81	33.15 ± 0.54
Soreness (mm)	Baseline	3 ± 3	11 ± 9		1 h	33.97 ± 1.06	33.16 ± 0.56
	2 h*	38 ± 41	50 ± 49		2 h^$^	32.89 ± 0.97	32.28 ± 0.72
	24 h*	52 ± 53	93 ± 40	IMT at 2 cm (°C)*^$^	Baseline	35.06 ± 0.73	34.81 ± 1.11
MMP-9 (ng.mL^−1^)	Baseline	644 ± 242	802 ± 378		P-ex*	36.66 ± 0.96	36.28 ± 0.52
	2 h*	1,031 ± 368	1,278 ± 349		P-int^$^	36.60 ± 0.56	34.98 ± 0.42
	24 h	536 ± 134	626 ± 307		1 h^$^	35.42 ± 0.59	34.39 ± 0.69
hs-CK (U.L^−1^)	Baseline	379 ± 324	352 ± 277		2 h^$^	34.22 ± 0.88	33.79 ± 0.82
	24 h*	1,167 ± 1,285	823 ± 668	IMT at 3 cm (°C)*^$^	Baseline	35.95 ± 0.67	35.85 ± 0.76
CRP (mg.L^−1^)	Baseline	1.2 ± 1.0	1.2 ± 1.1		P-ex*	37.73 ± 0.38	37.45 ± 0.38
	24 h	0.9 ± 0.6	1.7 ± 1.7		P-int*^$^	37.20 ± 0.31**	36.12 ± 0.26
IL-6 (pg.mL^−1^)	Baseline	0.9 ± 0.5	1.3 ± 0.8		1 h^$^	36.30 ± 0.42**	35.55 ± 0.41
	2 h*	1.4 ± 0.5	2.5 ± 1.5		2 h^$^	35.30 ± 0.70	35.16 ± 0.48
	24 h	1.4 ± 1.1	2.2 ± 2.2				

HWI, hot water immersion; PAS, passive recovery group; MVIC, maximum voluntary contraction; MMP-9, matrix metalloproteinase-9; CK, creatine kinase; hs-CRP, high sensitivity C-reactive protein; IL-6, interleukin-6; IMT, intramuscular temperature.

*significantly (*p* < 0.05) different from baseline (main effect of time); $ significantly (*p* < 0.05) different from post-exercise (main effect of time); ** significantly (*p* < 0.05) different from PAS; *$ significant (*p* < 0.05) difference between PAS, and HWI (main effect of group).

### MVIC

**FIGURE 2:**
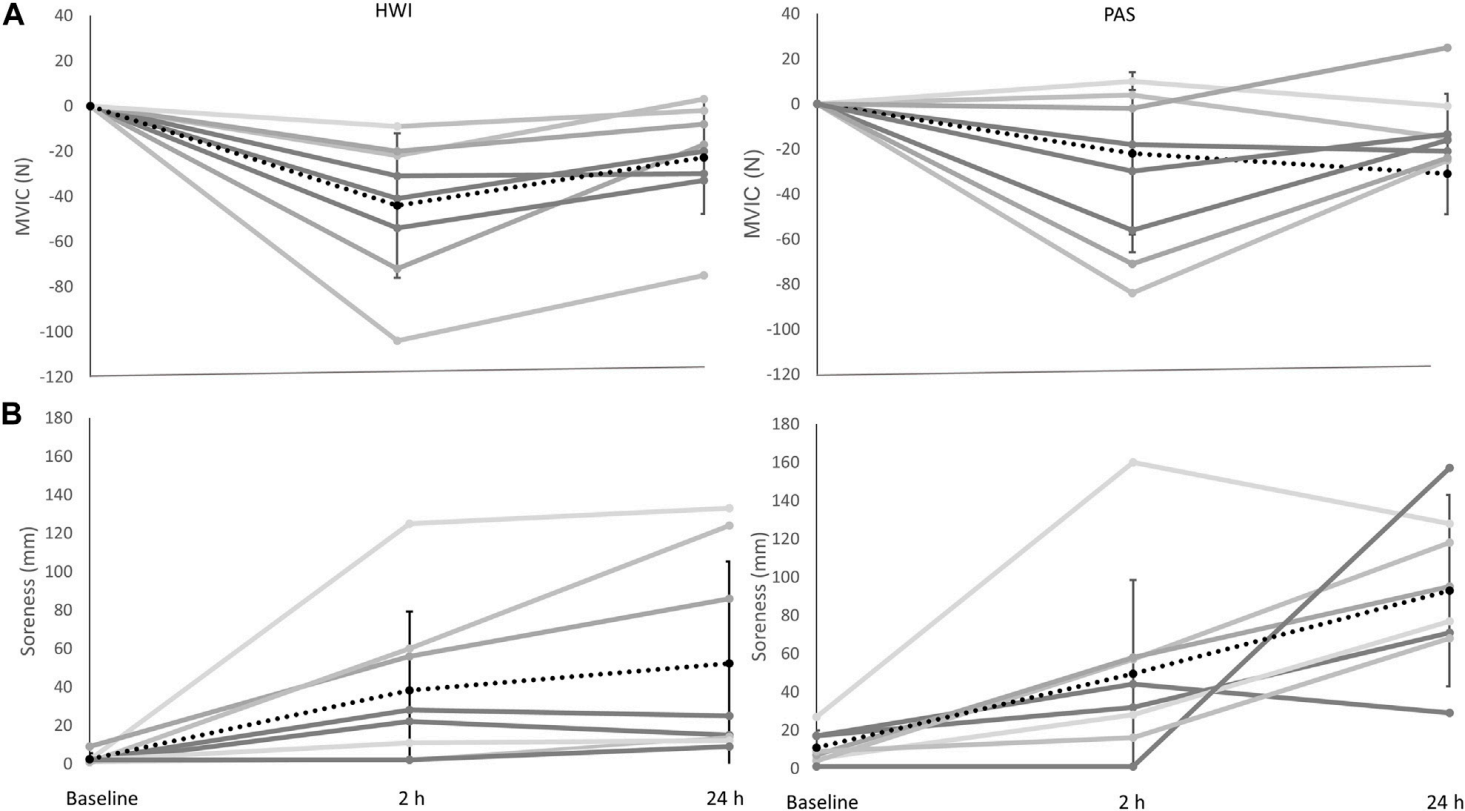
**(A)** Change in MVIC in the passive recovery (PAS) and hot water immersion (HWI) group (from left to right). **(B)** Change in muscle soreness in PAS and HWI (from left to right). Black dashed line represents the corresponding group average across time and standard deviation; grey lines represent individual responses across time. Significant differences (*p* < 0.05) between baseline, and 2 h and 24 h were evident for both MVIC and muscle soreness (main effect of time). Note: for MVIC the value shown at 2 h post is the change from baseline to 2 h post, and the value shown at 24 h is the change from baseline to 24 h.

There was a significant main effect of time (F_1.348, 18.870_ = 13.005, *P* = <0.000, ηp^2^ = 0.482), and *post hoc* pairwise comparisons determined significant decreases in MVIC between baseline-2 h, and −24 h post resistance exercise. There was no significant interaction effect (F_1.348, 18.870_ = 0.041, *p* = 0.904, ηp^2^ = 0.003) or main effect of group (F_1,14_ = 0.347, *p* = 0.565, ηp^2^ = 0.024) (Figure 2A). Comparisons for the group difference in the change from baseline to 2 h post (−14 N, 95%CI [-51, 22], *d* = 0.4) and baseline to 24 h (−9 N, 95% CI [-32, 14], *d* = 0.4) were unclear.

### Active muscle soreness

There was a significant main effect of time (F_2,28_ = 17.632, *P* = <0.001, ηp^2^ = 0.557) with *post hoc* comparisons showing significant increases in soreness from baseline-2 h, and −24 h after resistance exercise. No significant interaction (F_2,28_ = 1.287, *p* = 0.292, ηp^2^ = 0.084) or group effect (F_1,14_ = 2.064, *p* = 0.173, ηp^2^ = 0.128) was determined (Figure 2B). Comparisons for the group difference in the change from baseline to 2 h post (−3 mm, 95% CI [-48, 42], *d* = 0.07) and baseline to 24 h (−32 mm, 95% CI [-84, 20], *d* = 0.7) were unclear.

### MMP-9

Changes in MMP-9 were significant over time (F_2,28_ = 33.777, *P* = <0.001, ηp^2^ = 0.707) with *post hoc* comparisons identifying significant increases between baseline and 2 h post resistance exercise. There was no significant interaction (F_2,28_ = 0.582, *p* = 0.565, ηp^2^ = 0.040) or main (F_1,14_ = 1.620, *p* = 0.224, ηp^2^ = 0.104) effect of group. Comparisons for the group difference in the change from baseline to 2 h post (−98 ng mL^−1^, 95% CI [-410, 215], *d* = 0.3) and baseline to 24 h (81 ng mL^−1^, 95% CI [-209, 370], *d* = 0.3) were unclear.

### CK

Due to high baseline values in two participants (one per group) data were analysed for seven participants per group. There were significant increases between baseline and 24 h in CK (F_1,12_ = 56.402, *p* = <0.001, ηp^2^ = 0.825). There was no significant interaction effect (F_1,12_ = 0.189, *p* = 0.672, ηp^2^ = 0.015) or main effect of group (F_1,12_ = 0.039, *p* = 0.848, ηp^2^ = 0.003). There were no clear differences for group comparisons between baseline and 24 h (316 U.L^−1^, 95% CI [-2,967, 3,599], *d* = 0.1).

### Hs-CRP

Due to a high baseline value in one participant in the PAS group, data were analysed as seven (PAS) versus eight (HWI). There were no significant main effects of time (F_1,13_ = 1.339, *p* = 0.268, ηp^2^ = 0.093) or group (F_1,13_ = 0.260, *p* = 0.618, ηp^2^ = 0.020) and no significant interaction effect (F_1,13_ = 2.312, *p* = 0.152, ηp^2^ = 0.151). Group comparisons between baseline and 24 h were unclear (−0.8 mg.L^−1^, 95% CI [-2.0, 0.4], *d* = 0.8).

### IL-6

Due to high baseline values in two participants (one per group) data were analysed for seven participants per group. There was a significant main effect of time (F_2,24_ = 4.783, *p* = 0.018, ηp^2^ = 0.285) with *post hoc* tests determining a significant increase between baseline and 2 h post. There was no significant interaction effect (F_2,24_ = 0.093, *p* = 0.911, ηp^2^ = 0.008) or main effect of group (F_1,12_ = 1.286, *p* = 0.279, ηp^2^ = 0.097). Effects were unclear for group comparisons between baseline and 2 h post (−0.7 pg mL^−1^, 95%CI [-1.8, 0.4, *d* = −0.8), and baseline and 24 h (−0.3 pg mL^−1^, 95%CI[-2.1, 1.5), *d* = −0.2).

### Intramuscular temperature

There were significant main effects of time for intramuscular temperature (IMT) at 1 cm (F_2.232, 31.250_ = 9.144, *p* = <0.001, ηp^2^ = 0.395), 2 cm (F_1.866, 26.124_ = 29.305, *p* = <0.001, ηp^2^ = 0.677) and 3 cm (F_4,56_ = 60.753, *p* = <0.001, ηp^2^ = 0.813). Post-exercise resulted in the highest IMT at all depths, with significant increases at this point from baseline at 2 cm and 3 cm, and significant decreases from post-exercise to post-intervention (2 and 3 cm), 1 h post (2 and 3 cm) and 2 h post (all depths). Significant increases from baseline to intervention were only observed at 3 cm.

There were main effects of group at all IMT depths (1 cm: F_1,14_ = 9.758, *p* = .007, ηp^2^ = 0.411; 2 cm: F_1,14_ = 11.866, *p* = 0.004, ηp^2^ = 0.459; 3 cm: F_1,14_ = 10.321, *p* = 0.006, ηp^2^ = 0.424) with HWI eliciting higher intramuscular temperatures versus PAS.

There were no significant interaction effects at 1 cm (F_2.232, 31.250_ = 1.745, *p* = 0.188, ηp^2^ square = 0.111) or 2 cm (F_1.866, 26.124_ = 2.735, *p* = 0.087, ηp^2^ = 0.163). However, at 3 cm there was a significant interaction effect (F_4,56_ = 3.419, *p* = 0.014, ηp^2^ = 0.196). Post-hoc tests showed that at both post-intervention and 1 h post intramuscular temperature in HWI was significantly greater than PAS, furthermore between baseline and post-intervention there was only a significant increase in HWI.

Comparisons for the group differences are shown in Table 4. All effects were unclear except between baseline and post-intervention HWI resulted in a greater increase in intramuscular temperature compared to PAS at all depths.

## Discussion

The purpose of this study was to investigate the effect of HWI on acute physiological responses to resistance exercise. The results of this study indicate that HWI is a viable method of heat therapy that can maintain the rise in intramuscular temperature following resistance exercise. Despite this, there were no effects of HWI on measures of muscle function, muscle soreness or blood markers of muscle cell disruption or inflammatory processes. These results represent the first investigation into the acute physiological responses of a “real-world” HWI protocol following resistance exercise, alongside the use of a trained cohort, applied exercise session, and utilising good nutritional practice.

This is the first study to investigate the effects of HWI on intramuscular temperature during recovery from resistance exercise. After the post-exercise elevation in intramuscular temperature for both groups, the PAS group saw a decline following the intervention, whilst the HWI group showed a second increase post-intervention. Immediately following the intervention, HWI had the greatest effect on intramuscular temperature. HWI was able to maintain a higher intramuscular temperature compared to PAS up until 1 h post exercise at a depth of 3 cm.

As was previously hypothesised (Wilcock et al., 2006), the HWI-induced increase in tissue temperature would be expected to induce peripheral vasodilation and a subsequent increase in muscle blood flow. In the acute post-exercise period, a reduction in muscle blood flow, associated with cold water immersion, has typically been viewed as beneficial for exercise recovery due to reductions in inflammation, oedema and pain (Lee et al., 2005). This viewpoint has been challenged by recent research showing cold water immersion to exert no influence on muscle inflammatory or cellular stress responses (Peake et al., 2016).

It has previously been suggested that increases in muscle blood flow facilitate increased permeability of cellular, lymphatic and capillary vessels (Wilcock et al., 2006), leading to greater clearance rates of inflammatory markers in the blood. Given this, it is surprising that none of the blood markers in this study displayed enhanced rates of clearance following HWI. Only two previous studies investigating HWI have collected inflammatory markers from the blood after exercise, and in line with the current study, neither reported any effect of HWI (Vaile et al., 2008; Pournot et al., 2011). A mixture of findings related to post-exercise HWI and the appearance of intramuscular proteins in the blood currently exists with evidence of: a reduction (Vaile et al., 2008), a rise (Viitasalo et al., 1995) and no effect (Pournot et al., 2011). The only study to find an enhanced clearance utilised multiple immersions for each day up to 72 h post-exercise (Vaile et al., 2008). It is therefore reasonable to suggest that like others, the single bout of HWI in this study did not enhance the clearance of markers in the blood.

Research has shown both passive heat therapy (Uehara et al., 2004; Kobayashi et al., 2005; Yoshihara et al., 2013), and heat therapy in addition to a mechanical stimulus (Goto et al., 2003; Kakigi et al., 2011) to upregulate key anabolic signalling pathways and protein expression. The increased intramuscular temperature *per se* may therefore exert direct effects on cell signalling, impacting both inflammatory and anabolic pathways, although further research is required to confirm this.

Despite the manipulation of intramuscular temperature that could potentially aid the recovery of muscle function, we found no effect of HWI on MVIC. These results may be partially attributed to a reduction in acute physiological responses seen here in comparison to those reported by Jackman et al. (2019) which would have reduced the likelihood for an effect of the intervention. For example, at 24 h post-exercise the reduction in MVIC was 13% in the study by Jackman et al. (2019) and 7.2% in the present study. A possible cause could be differences in participant cohort, whereby those in the study by Jackman et al. (2019) had a 1 RM of 1.4 x BM, whilst the participants in the present study demonstrated 1.8 x BM. However, strength is not the primary determinant of training status (Buckner et al., 2017) and participants were recruited from an identical inclusion/exclusion criteria. Another possible explanation is the addition in the present study of added nutritional control which included the provision of protein supplements in the acute post-exercise period. Previous research has demonstrated that consuming protein (in the form of branched chain amino acids) can attenuate reductions in muscle function in the post-exercise period in resistance trained individuals (Howatson et al., 2012). This may therefore explain the reduced response seen between Jackman et al. (2019) and the present study. However, best practice recommendations (Jäger et al., 2017) suggest that individuals consume protein in the post-exercise period and therefore, in line with the applied nature of this study, the inclusion of the nutritional control enhanced ecological validity.

Previous research has typically produced mixed results related to the effect of post-exercise heat therapy on the recovery of muscle function, with those in support showing beneficial effects on all-out exercise performance (Cheng et al., 2017), strength (Vaile et al., 2008) and power (Viitasalo et al., 1995), although the mechanisms are still undefined. Cheng et al. (2017) reported that mean power output was better maintained following endurance exercise when the upper limbs were heated (∼38°C) compared to control (∼33°C) or being cooled (∼15°C). The authors attributed this to higher rates of glycogen resynthesis with heating. It is unlikely that MVIC measured in the present study were limited by muscle glycogen stores and highlights the need to match post-exercise strategies to specific recovery demands (Minett and Costello, 2015). Participants in the study by Cheng et al. (2017) were heated to an intramuscular temperature of ∼38°C at a depth of 1.5 cm, which is greater than the post-intervention intramuscular temperature of 35.0°C and 36.6°C reported in the present study for 1 and 2 cm, respectively. Despite differences in exercise modality, those that have reported beneficial effects of HWI, have used water temperatures of 37°C–38°C and durations of 14–20 min (Viitasalo et al., 1995; Vaile et al., 2008). It is unlikely that these protocols would have elevated intramuscular temperatures to greater levels than the present study, therefore we speculate that the intramuscular temperatures we report would have been comparable to previous studies that have reported beneficial effects. This lends to the assertion that in this instance, recovery of muscle function during maximal strength tasks following resistance exercise is independent of short-term changes in intramuscular temperature associated with HWI. Although isometric muscle function testing is considered the gold standard for absolute strength testing, it cannot be used as a proxy for dynamic muscle actions. As such, the results presented here may not accurately reflect the impact of HWI on more sport specific or ecologically valid sporting actions, and further research is warranted to investigate the utility of acute HWI for recovery in performance settings.

Our finding that HWI exerted no positive effect on ratings of muscle soreness is consistent with several reports (Kuligowski et al., 1998; Vaile et al., 2008; Pournot et al., 2011). Studies investigating heat therapy that have demonstrated beneficial effects have been limited to those that included underwater massage alongside HWI (Viitasalo et al., 1995) or continuous (8 h) low-level heat wrap therapy (Mayer et al., 2006) and would therefore be expected to elicit a markedly different response to HWI in the present study. Heat therapy has been suggested to reduce pain via an analgesic effect on nerves (Baker et al., 2001), although this theory has been proposed within the field of physiotherapy. Together, these results suggest that for those interested in applying HWI following resistance exercise, it is unlikely to produce reductions in muscle soreness.

A number of points are worth considering when interpreting the results of the present study. Firstly, a passive recovery protocol was employed as the comparator to HWI. Although it is unlikely that athletic individuals will employ a sedentary post-exercise strategy, to understand the effect of HWI on acute physiological responses, it is recommended to compare with no recovery method (White and Caterini, 2017). Secondly, it is important to understand the implications of our findings in the context of the study. We recruited trained individuals, employed an applied exercise modality, delivered a realistic HWI protocol and utilised good nutritional practice around the session to enhance the ecological validity of our findings. We do not rule out the possibility that the influence of heat therapy on physiological responses may differ in other situations. For example, passive heat acclimation has been shown to improve muscle contractile properties which may have clinical relevance for individuals unable to exercise (Racinais et al., 2016). Lastly, the authors acknowledge that the relatively small sample size could have influenced the findings, particularly given the large interindividual variation in certain dependent variables. Therefore, further research is warranted to verify the results presented herein.

In summary, this is the first study to demonstrate HWI as a viable means of heat therapy that can increase intramuscular temperature. We also offer insights into the effect of HWI on acute physiological responses in a real-world environment. In the context of the present study it appears that HWI is no more effective than passive recovery for the recovery of muscle function, soreness and markers of muscle cell disruption and inflammation following resistance exercise. Further research is required to investigate the application of HWI in other contexts including: following other exercise modalities, using different durations/temperatures of immersion, and when used chronically as part of a long-term training programme.

## Data Availability

The raw data supporting the conclusion of this article will be made available by the authors, without undue reservation.

## Abbreviations

CK, Creatine kinase; DXA, Dual-energy x-ray absorptiometry; ELISA, Enzyme-linked immunosorbent assay; hsCRP, High-sensitivity C-reactive protein; HWI, Hot water immersion; IL-6, Interleukin-6; MMP-9, Matrix metalloproteinase-9; MVIC, Maximal voluntary isometric contraction; µM, Micromolar; N, Newtons; PAS, Passive recovery; RFD, Rate of force development; RM, Repetition maximum; VAS, Visual analogue scale.
